# A tumor-promoting role for soluble TβRIII in glioblastoma

**DOI:** 10.1007/s11010-021-04128-y

**Published:** 2021-03-26

**Authors:** Isabel Burghardt, Judith Johanna Schroeder, Tobias Weiss, Dorothee Gramatzki, Michael Weller

**Affiliations:** Laboratory of Molecular Neuro-Oncology, Department of Neurology & Brain Tumor Center, University Hospital and University of Zurich, Frauenklinikstrasse 26, 8091 Zurich, Switzerland

**Keywords:** Betaglycan, Glioblastoma, Glioma, SMAD, TGF-β, TGF-βRIII (TβRIII)

## Abstract

**Purpose:**

Members of the transforming growth factor (TGF)-β superfamily play a key role in the regulation of the malignant phenotype of glioblastoma by promoting invasiveness, angiogenesis, immunosuppression, and maintaining stem cell-like properties. Betaglycan, a TGF-β coreceptor also known as TGF-β receptor III (TβRIII), interacts with members of the TGF-β superfamily and acts as membrane-associated or shed molecule. Shed, soluble TβRIII (sTβRIII) is produced upon ectodomain cleavage of the membrane-bound form. Elucidating the role of TβRIII may improve our understanding of TGF-β pathway activity in glioblastoma

**Methods:**

Protein levels of TβRIII were determined by immunohistochemical analyses and ex vivo single-cell gene expression profiling of glioblastoma tissue respectively. In vitro, TβRIII levels were assessed investigating long-term glioma cell lines (LTCs), cultured human brain-derived microvascular endothelial cells (hCMECs), glioblastoma-derived microvascular endothelial cells, and glioma-initiating cell lines (GICs). The impact of TβRIII on TGF-β signaling was investigated, and results were validated in a xenograft mouse glioma model

**Results:**

Immunohistochemistry and ex vivo single-cell gene expression profiling of glioblastoma tissue showed that TβRIII was expressed in the tumor tissue, predominantly in the vascular compartment. We confirmed this pattern of TβRIII expression in vitro. Specifically, we detected sTβRIII in glioblastoma-derived microvascular endothelial cells. STβRIII facilitated TGF-β-induced Smad2 phosphorylation in vitro and overexpression of sTβRIII in a xenograft mouse glioma model led to increased levels of Smad2 phosphorylation, increased tumor volume, and decreased survival

**Conclusions:**

These data shed light on the potential tumor-promoting role of extracellular shed TβRIII which may be released by glioblastoma endothelium with high sTβRIII levels.

**Supplementary Information:**

The online version contains supplementary material available at 10.1007/s11010-021-04128-y.

## Introduction

Glioblastoma is one of the most common malignant intrinsic brain tumors [1]. Prominent biological features of glioblastoma include excessive migratory, invasive and angiogenic potential, and suppression of anti-tumor immune surveillance. Glioma-derived transforming growth factor (TGF)-β is thought to be fundamental in these processes. Self-renewing, highly tumorigenic glioma-initiating cells (GIC) have been proposed to be more resistant to therapy than the tumor bulk [2], to have invasive properties and to be involved in angiogenesis [3–5]. TGF-β signaling has been proposed to be a key regulator in glioma vasculature [6] and the maintenance of stem cell-like properties and tumorigenic activity of GIC [7, 8]. Thus, targeting TGF-β may affect glioma vessels and GIC, thereby inhibiting tumor growth and sensitizing tumors to conventional therapies [9]. Proteins of the TGF-β superfamily interact with TGF-β receptors I–III (TβRI-III). TβRI, also known as activin receptor-like kinases (ALK), and TβRII form heteromeric complexes upon binding of TGF-β family ligands [10]. Subsequently, canonical and non-canonical TGF-β signal transduction is activated. Canonical TGF-β signal transduction is mediated by Smad transcription factors resulting in the phosphorylation of the receptor-regulated Smads, Smad 1, 2, 3, 5, and 8 [11]. TGF-β receptors may also directly interact with or phosphorylate non-Smad proteins initiating parallel signaling that cooperates with the Smad pathway in downstream responses [12]. The TGF-β receptor core complex is not only constituted by TβRI and TβRII, but also by the TGF-β signaling coreceptor TGF-β receptor type III (TβRIII), a ubiquitously expressed accessory TGF-β receptor [13]. TβRIII is a transmembrane protein with a large extracellular domain (ECD) with glycosaminoglycan groups [41]. By its ECD, it may bind multiple members of the TGF-β family such as TGF-β1-3, activin-A, bone morphogenetic proteins (BMP)-2, BMP-4, BMP-7, and growth differentiation factor (GDF) 5 as well as inhibin [14, 15]. TβRIII undergoes ectodomain shedding from the cell surface to generate soluble forms of the receptor [16]. The ECD is then capable of binding TGF-β ligands [17]. Overall, the role of TβRIII is complex and difficult to predict as the balance of cell surface and shed TβRIII, which is also called soluble TβRIII (sTβRIII), may differentially regulate TGF-β superfamily signaling [13, 18]. Given the putative central role of TGF-β superfamily signaling in glioblastoma, the present study focused on the role of TβRIII in the regulation of TGF-β pathway activity in this disease.

## Material and methods

### Cell culture and reagents

Details are summarized in supplementary note 1.

### Real-time PCR (RT-PCR)

RT-PCR was performed as previously described [19] and is described in supplementary note 2.

### Immunoblot analyses

Immunoblot analysis was performed as previously described [20], and reagents are listed in supplementary note 1.

### Enzyme-linked immunosorbent assay (ELISA)

Experimental details are listed in supplementary note 3.

### Flow cytometry

LTCs were seeded at confluency and flow cytometry was performed 48 h after serum deprivation [19].

### Animal studies

Experimental details are described in supplementary note 4.

### Immunohistochemistry

Experimental steps were conducted as described in supplementary note 5.

### Immunofluorescence microscopy

Experimental details are provided in supplementary note 6.

### Single-cell real-time polymerase chain reaction (scRT-PCR) of reverse-transcribed RNA

Experimental details are described in supplementary note 7.

### Statistical analysis

Details are provided in supplementary note 8.

## Results

### TβRIII expression in gliomas in vivo

**Fig. 1:**
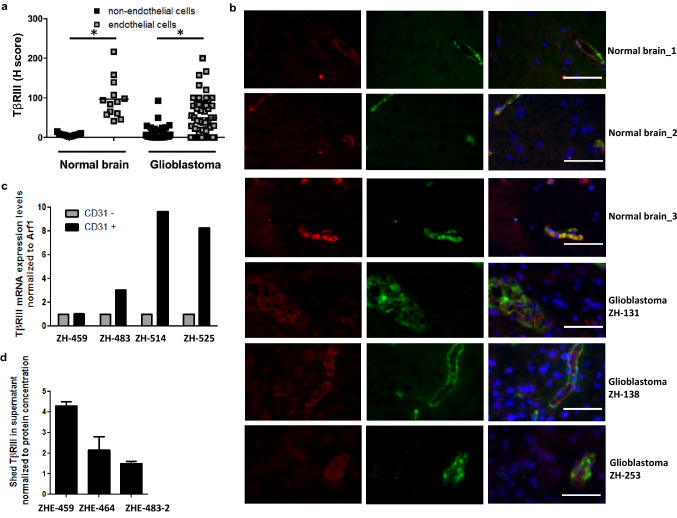
TβRIII levels in human glioblastoma in vivo. **a** TβRIII was detected immunohistochemically and quantified using the H-score in a TMA comprising 13 normal brain tissue samples obtained from epilepsy surgery and in tissue sections of 57 newly diagnosed and nine recurrent glioblastoma patients. Non-endothelial and endothelial cells, which were identified topographically aligning tumor vessels, were analyzed and scored separately; mean values are marked by the line, *indicates significance *p* < 0.001. **b** Representative photomicrographs of tissue sections from **a** analyzed for expression of TβRIII (red) and the endothelial marker vWF (green) by immunofluorescence. Scale bar: 50 µm. DNA was stained with 4′,6-diamidino-2-phenylindole (DAPI). **c** TβRIII expression was determined in CD31^+^ versus CD31^−^ cell fractions of freshly dissociated glioblastoma tissues by RT-PCR. **d** Supernatants from CD31^+^ (endothelial) cells of three freshly dissected glioblastomas were assessed by ELISA for levels of shed TβRIII. Data were normalized to protein concentration of supernatants. (Color figure online)

Immunohistochemistry was used to assess TβRIII levels in normal human brain and in glioblastoma tissue samples. Normal brain of 13 individuals who were not diagnosed with a glioblastoma was tested using a tissue microarray (TMA). The TMA included normal brain hippocampus tissue punches of 12 different individuals and three punches of a gyrus temporo-occipitalis tissue of one individual. TβRIII in glioblastoma was examined in 52 newly diagnosed and nine recurrent tissue samples (Supplementary Table 1). TβRIII protein levels were analyzed separately in the tumor and normal brain tissue versus the endothelium. Quantitative assessment of non-endothelial regions of tumor and normal brain tissue separately from the respective endothelial regions revealed higher TβRIII levels in the vasculature of glioblastoma (*p* < 0.001) and of normal brain (*p* < 0.001) than in the tumor tissue proper or the parenchymal brain cells (Fig. 1a). Representative paraffin sections of four glioblastoma and four normal brain patients stained for TβRIII are shown in Figure S1. Survival data were available for all 52 glioblastoma patients assessed for TβRIII staining in the tumor cells and for 48 patients assessed for TβRIII staining in the endothelial cells. Comparison of the median overall survival (OS) of glioblastoma patients with high versus low TβRIII levels (cut-off defined by median TβRIII levels) revealed no differences, neither for patients with high (median OS 17.7 months, 95% confidence interval (CI) 11.8–23.5 months) versus low (median OS 15.8 months, 95% CI 5.8–25.8 months) staining in the tumor cells (*p* = 0.88) (Figure S2A) proper nor for patients with high (median OS 19.0 months, 95% CI 8.3–29.7 months) versus low (median OS 17.7 months, 95% CI 0.0–53.5 months) staining in the glioblastoma vasculature (*p* = 0.36) (Figure S2B).

Representative normal brain and glioblastoma tissue sections with the endothelial marker vWF and TβRIII stained by co-immunofluorescence confirmed a predominant vascular localization both within the normal brain and the glioblastoma sections (Fig. 1b). TβRIII levels in tumor cells positively correlated with TβRIII levels in endothelium in newly diagnosed (*r* = 0.412, *p* = 0.004) as well as in recurrent glioblastoma (*r* = 0.818, *p* < 0.005). Regarding correlations of TβRIII with expression of other TGF-β related molecules in this glioblastoma patient cohort, only a correlation in newly diagnosed glioblastoma (tumor cells) of protein levels of TβRIII and TGF-β1 (*r* = 0.339, *p* = 0.046) and in recurrent glioblastoma (tumor cells) of protein levels of TβRIII and pSmad1/5/8 (*r* = 0.898, *p* = 0.033) were detected (Supplementary Table 2). In a previous study, we found a localization of CD31 restricted to the tumor blood vessels in glioblastomas [21], defining CD31 as an endothelial marker in glioblastoma. As a confirmation that TβRIII is more abundant in tumor endothelium than in the tumor cells, we used the endothelial cell marker CD31 and compared matched CD31^+^ and CD31^−^ cell populations from four freshly dissociated primary glioblastoma tissues. In three out of four tumors, TβRIII expression was higher in the CD31^+^ cell fraction (Fig. 1c). Endothelial-like (CD31^+^) cell lines previously established in our laboratory [22] also released sTβRIII into the supernatant (Fig. 1d).

## Single-cell analysis of TβRIII expression in freshly glioma cells ex vivo

**Fig. 2:**
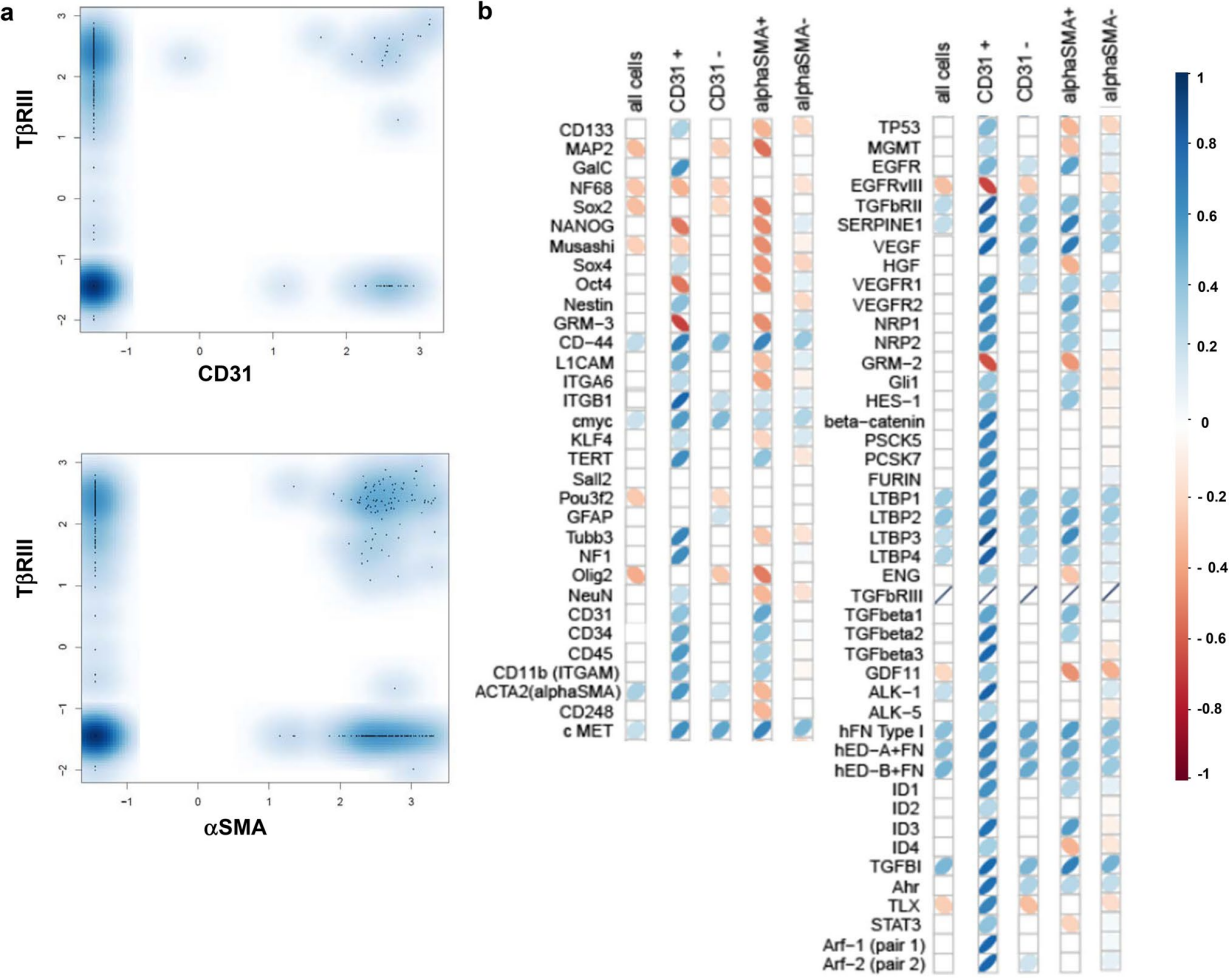
TβRIII expression determined by single-cell RT-PCR in freshly dissociated human glioblastomas. **a** Distribution of TβRIII expression in CD31- or αSMA-negative versus positive populations as determined by single-cell RT-PCR in cells derived from freshly dissociated human glioblastoma (*N* = 6, pooled). *x*-axis: normalized relative log2 expression value of CD31 (upper panel) and αSMA- (lower panel), *y*-axis: normalized relative log2 expression value of TβRIII. **b** Correlation matrix between TβRIII expression and indicated genes on a single-cell level in all cells and in the CD31-negative- versus the CD31-positive subpopulations. Pearson correlation coefficients are visualized using red or blue tilted symbols, indicating negative or positive correlations, respectively. Only significant correlations with *p*-values < 0.05 (after Bonferroni correction) are shown

Next, we expanded our differentiated analysis of TβRIII in the tumor versus vascular compartment to the single-cell level in cells from glioblastoma tissue obtained directly from surgery. To monitor and quantify the heterogeneity of TβRIII mRNA in these single cells, we evaluated single-cell profiles of six different patients. We performed single-cell real-time polymerase chain reaction (scRT-PCR) of TβRIII, and of CD31 or alpha-smooth muscle actin (αSMA), as markers of the endothelial and pericytic/vascular smooth muscle cells (VSMCs). Of the 481 single cells analyzed, 43 cells expressed CD31 and 185 cells expressed αSMA (Figure S3). TβRIII mRNA was expressed in 156 of 438 cells (36%) of the CD31-negative versus 21 of 43 cells (56%) of the CD31-positive cells, and in 84 of 296 cells (28%) of the αSMA-negative versus 93 of 185 cells (50%) of the αSMA-positive cell population. This confirmed the higher proportion of TβRIII-expressing cells in the vascular compartment. Yet, remarkably, one third of the CD31-negative cells expressed TβRIII, too (Fig. 2a). Correlation analysis of all 481 cells of the six glioblastoma patients on single-cell level revealed only weak correlations of TβRIII mRNA expression with molecules associated with TGF-β superfamily signaling such as the extracellular matrix proteins LTBP (LTBP-1, *r* = 0.2, *p* = 0.00036, LTBP-2, *r* = 0.18, *p* = 0.00005, LTBP-3, *r* = 0.17, *p* = 0.014012, LTBP-4, *r* = 0.17, *p* = 0.0017), fibronectin (FN) (*r* = 0.18, *p* = 0.000003) and its oncofetal isoforms EDA + FN (*r* = 0.21, *p* = 0.000018) and EDB + FN (*r* = 0.25, *p* = 0.000002), and the TGF-β target gene TGF-β induced (TGFBI) (*r* = 0.23, *p* = 0.000004), and remarkably no correlation with the three TGF-β isoforms. Analysis of subpopulations specific for tumor vasculature, such as the CD31-positive and αSMA-positive subpopulations, revealed for both subpopulations additional positive correlations of TβRIII mRNA expression with molecules associated with TGF-β superfamily signaling such as all three TGF-β isoforms, TGF-βRII, the TGF-β target gene serpine1 and molecules associated with angiogenic signaling such as VEGF, and associated receptors such as vascular endothelial growth factor receptor (VEGFR)1, VEGFR2, neuropilin (NRP)1, and NRP2. In the CD31-positive subpopulation, there was a positive correlation of TβRIII and further molecules linked to TGF-β signaling such as ALK-1, aryl hydrocarbon receptor (AhR), the proTGF-β processing enzymes proprotein convertase subtilisin/kexin type (PCSK) 5, PCSK 7 and furin, and the TGF-β target genes Id (inhibitor of DNA binding) 1 and Id3 (Fig. 2b).

## TβRIII expression and release in human glioma cell lines

**Fig. 3:**
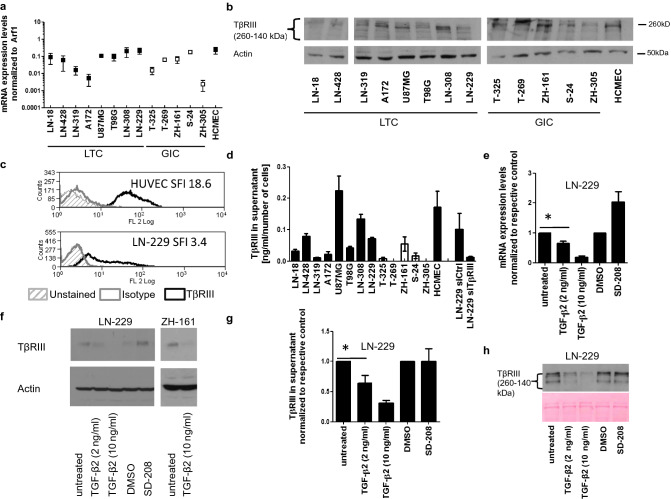
TGF-β downregulates TβRIII in human glioma cell lines. **a** RT-PCR analysis of TβRIII mRNA expression in human LTC (LN-18, LN-428, LN-319, A172, U87MG, T98G, LN-308, LN-229), GIC (T-325, T-269, ZH-161, S-24, ZH-305), and hCMEC normalized to Arf1. **b** TβRIII protein levels in total cell lysates were examined by immunoblot, and actin was used as a loading control. **c** Cell surface TβRIII protein levels were assessed by flow cytometry in LN-229 cells, HUVEC cells were measured as positive control. Protein levels are expressed as mean specific fluorescence index (SFI). **d** Shed TβRIII levels normalized to the total cell number of the respective supernatants were determined by ELISA. A transient knockdown of TβRIII in LN-229 cells was included as a negative control. **e** Relative changes of TβRIII mRNA expression in LN-229 cells were assessed after exposure to TGF-β2 (2 or 10 ng/ml) or to SD-208 (5 µM) for 24 h. Expression ratios relative to the respective control are depicted. **f** TβRIII protein levels in whole cell lysates of LN-229 cells were detected by immunoblot following the treatments described in **e**. **g** and **h** Shed TβRIII levels in LN-229 glioma cell supernatants following the treatments as described in **e** were determined by ELISA (**g**) and by immunoblot (**h**), ponceau S staining is shown as loading control in **h**

We assessed TβRIII levels in a panel of human long-term cell lines (LTC), glioma-initiating cell (GIC) cultures, and cultured human brain-derived microvascular endothelial cells (HCMEC). TβRIII was consistently expressed in LTC and GIC on mRNA (Fig. 3a) and protein levels as assessed in total cellular lysates (Fig. 3b). By flow cytometry, we ensured the localization of TβRIII on the cell surface (Fig. 3c). Moreover, sTβRIII was detected in the supernatant (Fig. 3d) at levels correlating with the levels in cellular lysates, indicating constitutive shedding of the receptor in the cell lines investigated (*r* = 0.69; *p* = 0.0059). Next, we evaluated the effect of rhTGF-β2 and of TGF-β signaling blockade using the TGF-βRI inhibitor SD-208 [23]. Upon TGF-β2 stimulation, TβRIII mRNA expression was reduced in a concentration-dependent manner in LN-229 cells. Conversely, abrogation of TGF-βRI signaling by SD-208 increased TβRIII mRNA expression (Fig. 3e). Similar changes were seen on protein level in cell lysates of LN-229 and ZH-161 cells (Fig. 3f). TβRIII levels in the supernatant were decreased by TGF-β, too but not increased by SD-208 (Fig. 3g,h).

## Modulation of TGF-β signaling by (soluble) TβRIII

**Fig. 4:**
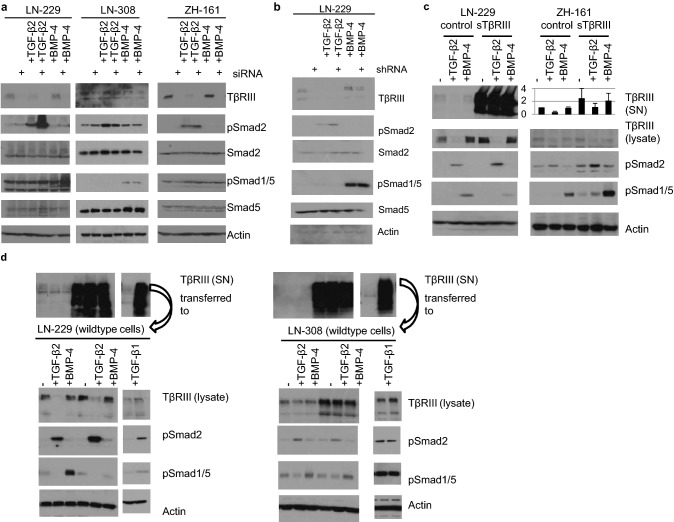
sTβRIII increases pSmad2 signaling. **a** LTCs (LN-229 and LN-308) or GIC (ZH-161) were transfected with a non-targeting control or siRNA pools targeting TβRIII; 48 h after siRNA transfection, the cells were stimulated with BMP-4 (1 ng/ml) or TGF-β2 (2 ng/ml) for 24 h. **b** LN-229 cells stably transfected with pcDNA control shRNA or TβRIII-specific shRNA were serum starved for 24 h and subsequently stimulated with 1 ng/ml BMP-4 or 2 ng/ml TGF-β2 for 24 h. **c** LN-229 or ZH-161 cells stably overexpressing sTβRIII or respective control cells were serum-starved for 24 h and subsequently stimulated with 2 ng/ml TGF-β2 or 1 ng/ml BMP-4 for 24 h. Shed TβRIII levels were assessed in cell culture supernatants by immunoblot for LN-229 or ELISA for ZH-161 cells. **d** Cell culture supernatants of 24 h serum-starved LN-229 cells lentivirally overexpressing TβRIII (LentiORF TβRIII cells) or the respective control cells (LentiORF-control cells) were transferred onto the indicated wildtype cells (LN-229, panels on the left; LN-308, panels on the right), for 24 h with additional stimulation with 2 ng/ml TGF-β2, 1 ng/ml BMP-4 or 2 ng/ml TGF-β1. Shed TβRIII levels of the respective aliquots of these cell culture supernatants are visualized by immunoblot analysis (upper panels). **a**–**d** For all samples, whole cell lysates were assessed for TβRIII, pSmad2, Smad2, pSmad1/5, Smad5, or actin protein levels by immunoblot

To explore the role of TβRIII in TGF-β superfamily signaling, we transiently silenced its expression in LN-229, LN-308, or ZH-161 cells and studied their response to TGF-β superfamily ligand stimulation at the level of Smad phosphorylation. LN-229 cells transiently depleted of TβRIII by siRNA exhibited increased pSmad2 levels, but no changes in pSmad1/5 (Fig. 4a, left panel, lane 1 versus lane 2). In response to rhTGF-β2 or rhBMP-4, TβRIII-depleted LN-229 cells showed increased pSmad2 or pSmad1/5 levels, respectively (Fig. 4a, left panel, lane 3 versus lane 4 for TGF-β2 stimulation and lane 5 versus lane 6 for BMP-4 stimulation). In LN-308 and ZH-161 cells, transient TβRIII depletion by siRNA did not modulate pSmad2 or pSMAD1/5, neither on constitutive levels nor upon stimulation with exogenous TGF-β2 or BMP-4 (Fig. 4a, middle and right panel). Similar to the effects of the transient siRNA-mediated knockdown in LN-229 cells, stable lentivirus-based TβRIII depletion in LN-229 cells (shRNA) increased constitutive pSmad2 levels as well as pSmad2 levels upon TGF-β2 stimulation but did not affect pSmad1/5 either constitutively or upon BMP-4 stimulation (Fig. 4b).

To analyze the specific effects of sTβRIII, we overexpressed a mutated form of TβRIII comprising only the extracellular domain of TβRIII. This had no effect on baseline constitutive pSMAD levels, but increased pSmad2 levels in response to TGF-β2 in LN-229 cells (Fig. 4c, left panel lanes 2 versus 5); furthermore, increased baseline and TGF-β2-evoked pSmad2 levels were observed in ZH-161 cells overexpressing sTβRIII (Fig. 4c, right panel, lanes 2 versus 5 and 1 versus 4). Overexpression of sTβRIII reduced pSmad1/5 levels in LN-229 cells stimulated with BMP-4 (Fig. 4c, left panel, lanes 3 versus 6), whereas baseline and TGF-β2-or BMP-4-evoked pSmad1/5 levels were increased in ZH-161 cells overexpressing sTβRIII (Fig. 4c, right panel, lanes 3 versus 6). For confirmation, we transferred supernatants of LN-229 cells overexpressing full-length TβRIII (LentiORF-TβRIII cells) or of the respective control cells (LentiORF-control cells) onto wildtype glioma cells. In line with our results from the overexpression approach of sTβRIII shown in Fig. 4c, the supernatants of the cells overexpressing TβRIII activated TGF-β2/Smad2 signaling in wildtype LN-229 cells when stimulated with TGF-β2 (Fig. 4d, left panel, lane 2 versus lane 5) and reduced pSmad1/5 levels when stimulated with BMP-4 (Fig. 4d, left panel, lane 3 versus lane 6). To investigate a possible differential modulating effect of sTβRIII on TGF-β1- versus TGF-β2-mediated signaling, we costimulated with TGF-β1 when supernatant from LN-229 cells with shed TβRIII was transferred onto wildtype LN-229 cells. Still, an increase in pSmad2 also occurred when costimulation with TGF-β1 was performed (Fig. 4d). In LN-308 shed, TβRIII did not modulate Smad-dependent signaling when we transferred supernatant with high levels of shed TβRIII onto this cell line (Fig. 4d, right panel), consistent with the lack of effect when TβRIII was depleted (see Fig. 4a).

## (Soluble) TβRIII promotes experimental glioma growth in vivo

**Fig. 5:**
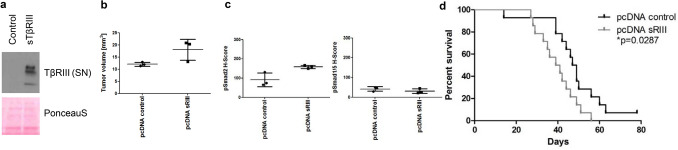
Overexpression of sTβRIII in a xenograft mouse model. **a** TβRIII expression in cell culture supernatants (Ponceau S staining as loading control) of ex vivo cell cultures generated from established tumors from athymic CD1 nude mice (LN-229 pcDNA control versus LN-229 pcDNA sTβRIII) detected by immunoblot. **b** Tumor volume from established tumors from athymic CD1 nude mice (LN-229 pcDNA control versus LN-229 pcDNA sTβRIII) calculated as length*width*depth multiplied by π/6 of 3 animals. **c** H scores from established tumors from athymic CD1 nude mice (LN-229 pcDNA control versus LN-229 pcDNA sTβRIII) for pSmad2 (left panel) and pSmad1/5 (right panel). **d** Overall survival data for CD1 nude mice inoculated intracerebrally with 75,000 LN-229 pcDNA control versus LN-229 pcDNA sTβRIII presented as a Kaplan–Meier plot

Finally, we studied whether the biological effects of altered TβRIII availability translated into altered glioma growth in vivo. In vitro*,* LN-229 overexpressing sTβRIII showed no differences in proliferation or clonogenicity (data not shown). LN-229 cells stably overexpressing sTβRIII were implanted into the right striatum of athymic CD1 nude mice. Cell lines generated ex vivo from mice-bearing LN-229 sTβRIII or control tumors at the time of sacrifice confirmed stable overexpression of sTβRIII (Fig. 5a). Assessment of tumor volumes of three mice of each group when the first clinical symptoms occurred showed increased tumor volumes (Fig. 5b) and increased pSmad2 levels in the tumors whereas pSmad1/5 levels were unaffected (Fig. 5c). Survival of nude mice with orthotopically implanted tumors overexpressing sTβRIII was significantly decreased (Fig. 5d).

## Discussion

TGF-β and its receptors TβRI and TβRII have been well studied in glioblastoma [24, 25]; however, little is known about the coreceptor TβRIII. TβRIII is ubiquitously expressed on most cell types, however, commonly not on endothelial cells [26]. TβRIII is commonly considered a tumor suppressor [26], since its expression is lost during progression of several tumor entities, e.g., breast cancer [27], non-small cell lung cancer [28], and prostate cancer [29]. Sequestering of TGF-β by shed TβRIII inhibiting downstream signaling may be a mechanism of action [13, 18, 27, 28, 30]. However, a tumor-promoting role for TβRIII has been deduced from the reduction of tumorigenicity when TβRIII expression was reduced in metastatic breast cancer cells [31] and in human breast cancer stroma [32]. In high-grade non-Hodgkin`s lymphomas [33], colon cancer [34], and B-cell chronic lymphatic leukemia [35], TβRIII expression is increased and not lost during cancer progression and may increase tumorigenicity [34]. A dichotomous role of TβRIII has been described in the context of lung cancer where an extracellular mutant of TβRIII with enhanced ectodomain shedding reduced tumorigenicity of the respective tumor cells but increased their growth rate in vitro and in vivo [36]. A study revealed reduced levels of TβRIII in the tumor stroma. Further, there were distinct paracrine roles of sTβRIII in the tumor microenvironment depending whether it was derived from normal or cancer tissue what emphasizes the complex regulation of availability of cytokines in the ECM [32]. Gene therapy with sTßRII and sTßRIII pointed towards a possible therapeutic role of sTβRII and sTβRIII [37], what led us to perform the current profound study on TβRIII in glioblastoma.

TβRIII levels in newly diagnosed glioblastoma samples and of normal brain showed predominantly endothelial localization (Fig. 1a, b). Analysis of freshly dissociated glioblastoma tissues revealed higher mRNA levels in the endothelial fraction of the tumors (Fig. 1c), and endothelial cell lines isolated from freshly dissected glioblastoma samples also secreted the shed form of TβRIII (Fig. 1d). Single-cell RT-PCR from freshly dissociated tissue of six glioblastoma patients confirmed this prevalence in the vascular compartment. Both in the vascular (CD31- and αSMA-positive cells) and in the non-vascular compartment, TGF-βRIII correlated with molecules associated with TGF-β superfamily signaling such as LTBP and FN which are both involved in the process of extracellular activation of TGF-β signaling [20, 21] (Fig. 2b). In line with previous studies on embryonic mouse cells [38], levels of cellular and shed TβRIII correlated in our cell line panel (Fig. 3b, d). As reported for ovarian and breast cancer cell lines [39], TGF-β negatively regulated TβRIII expression in a TβRI-dependent manner (Fig. 3e–h). Here, TGF-βRI inhibition increases membrane-associated TβRIII levels (Fig. 3f), but levels of sTβRIII were unaffected (Fig. 3g, h). In light of this finding, we speculate that in addition to negatively regulating TβRIII expression, TGF-β exerts a posttranscriptional effect on TβRIII by increasing shedding and, thus, increasing its release from the cell membrane. Interestingly, expression of TIMP2, an inhibitor of TβRIII shedding [17], is reduced in human glioma cell lines exposed to TGF-β2 [40].

In many models, TβRIII is described as a dual modulator of TGF-β signaling with cell surface TβRIII enhancing signaling and shed TβRIII acting as an antagonist by ligand sequestration [41]. More recently, shedding-independent inhibition of signaling exerted by the cytoplasmic domain, which sequestered type I and type II TGF-β receptors, has been described [42]. Depletion of TβRIII by transient (Fig. 4a) or stable (Fig. 4b) knockdown exerted an inhibitory effect on Smad signaling in the glioma cell line LN-229 as well.

To better distinguish effects of membrane associated versus soluble TβRIII, we overexpressed sTβRIII in LN-229 and ZH-161 cells. Surprisingly, sTβRIII activated Smad2 signaling in both models (Fig. 4c, d) and decreased survival in LN-229 tumor-bearing mice (Fig. 5). In the glioma cell line LN-308, we observed neither an effect on Smad signaling upon depletion of TβRIII (Fig. 4a) nor upon exposure to sTβRIII (Fig. 4d). Since LN-308 is a TGF-β-driven cell line as defined by high levels of furin, active TGF-β2, and pSmad2 [19, 43], it might not require additional coreceptor activity. Indeed, in a classical TGF-β activity assay with the TGF-β reporter cell line Mv1Lu (ATCC® CCL-64), sTβRIII enhances TGF-β bioactivity, too. Here, a fragment of sTβRIII comprising one fourth of the ECD which is closest to the membrane-spanning segment increased binding of TGF-β to TGF-β receptor II [44]. Similarly, soluble endoglin, a TGF-β type III receptor similar to TβRIII, does not inhibit TGF-β superfamily signal transduction but binds to circulating BMP-9 and induces signaling on endothelial cells [45]. There may be a strong dependency of TβRIII`s effects on the cellular context, e.g., presumably including the presence of other TGF-β superfamily ligands and receptors. Indeed, while sTβRIII inhibited BMP-4/Smad1/5 signaling in LN-229 cells, it activated BMP-4/Smad1/5 signaling in ZH-161 cells and did not modulate signaling in LN-308 cells (Fig. 4). The important role of the balance of shed and cell surface TβRIII for Smad1/5 signaling has been studied in breast cancer models [13, 18]. We observed an activating role for sTβRIII for Smad2 phosphorylation both in TGF-β1- and TGF-β2-mediated signaling in glioma cells (Fig. 4d). The potency of sTβRIII as a protumorigenic factor in glioma cells was confirmed in vivo using xenografts of LN-229 control versus LN-229 sTβRIII-overexpressing cells in nude mice. Orthotopically implanted sTβRIII-transfectants formed larger tumors (Fig. 5b) and had increased levels of pSmad2 in their tumors (Fig. 5c). In line, the animals had shorter survival compared to the controls (Fig. 5d). Previous work has pointed towards a role of TGF-β pathway activity in glioma vessels [6, 25, 46], and glioblastomas are one of the most vascularized tumors [24, 47, 48]. Endothelial cells might provide large amounts of sTβRIII within the tumor to promote TGF-β2/Smad2 signaling in glioma cells and thereby promote tumorigenicity. Our study may have implications for approaches of pharmacological targeting of TGF-β superfamily ligands using TGF-β binding traps derived from TGF-β (co-)receptors. Although systemic administration of a sTβRIII ECD has been shown to inhibit tumor growth in prostate and breast cancer models [49, 50], an opposite effect is indicated by our glioma model.

## Supplementary Information

Below is the link to the electronic supplementary material.

Supplementary file 1 (DOCX 71 kb)

Supplementary file 2 (PPTX 10466 kb)

## Abbreviations

GIC: Glioma-initiating cell lines
hCMEC: Human brain-derived microvascular endothelial cells
LTC: Long-term glioma cell lines
sTβRIII: Soluble TβRIII
TGF: Transforming growth factor
TβRIII: TGF-β receptor III
