# Supplementary material for: A tumor-promoting role for soluble TβRIII in glioblastoma
Source: Mol Cell Biochem. 2021 Mar 26;476(8):2963–73. doi: 10.1007/s11010-021-04128-y (PMC8263459; doi:10.1007/s11010-021-04128-y)
Supplement: Supplementary file 1 — (DOCX 71 kb) [file 11010_2021_4128_MOESM1_ESM.docx]

**Figure S1.** TβRIII was detected immunohistochemically in paraffin sections in glioblastoma specimens (A) and in the normal brain tissue samples from patients not suffering from a glioblastoma (B) using the TβRIII antibody AF-242-PB (R&D Systems) as primary antibody. Representative photomicrographs of four patients with glioblastoma and of four patients suffering from epilepsy (normal brain tissue) are shown. Scale bars: 50 µm.

**Figure S2.** Kaplan-Meier survival curves are shown for patients stratified by TβRIII expression levels; the median expression level was used as cut-off to define patients with high (black) or low expression (grey). Date are shown for the expression in (A) the tumor cells or (B) the endothelial compartment. The log-rank test was used for comparison.

**Figure S3.** Distribution of CD31 and aSMA expression in cells derived from freshly dissociated human glioblastoma (n=6). X-axis: CD31 expression value, y-axis: αSMA expression value.

**Supplementary table 1.** Patient characteristics

|  | **Newly diagnosed glioblastoma**  **n=52** | **Recurrent glioblastoma**  **n=9** |
| --- | --- | --- |
| Age*; years  Median  Range | 59.1  32-79 | 47.0  19-62 |
| Gender; n (%)  Female  Male | 22 (42.3)  30 (57.7) | 2 (22.2)  7 (77.8) |
| Karnofsky performance status*; n (%)  <70%  70-80%  90-100%  no data | 12 (23.5)  29 (56.9)  10 (19.6)  1 | 1 (12.5)  4 (50.0)  3 (37.5)  1 |
| Extent of resection; n (%)  biopsy  incomplete  gross total  no data | 2 (3.9)  34 (66.7)  15 (29.4)  1 | 0 (0.0)  5 (62.5)  3 (37.5)  1 |
| First-line therapy; n (%)  no therapy (beyond surgery)  radiotherapy alone  alkylating chemotherapy alone  radiotherapy plus temozolomide  no data | 1 (2.0)  13 (26.0)  2 (4.0)  34 (68.0)  2 | 0 (0.0)  2 (25.0)  0 (0.0)  6 (75.0)  1 |

*, at time of diagnosis or recurrence, respectively.

**Supplementary table 2.** Correlation analyses

|  | **TβRIII (H score)** | | | |
| --- | --- | --- | --- | --- |
|  | **newly diagnosed glioblastoma** | | **recurrent glioblastoma** | |
|  | **tumor cells** | **endothelial cells** | **tumor cells** | **endothelial cells** |
| **TGF-β1**  **(mRNA)** | r = 0.058  p = 0.738 | r = 0.035  p = 0.84 | r = -0.519  p = 0.245 | r = -0.894  p = 0.1 |
| **TGF-β2**  **(mRNA)** | r = 0.308  p = 0.072 | r = 0.113  p = 0.518 | r = -0.334  p = 0.471 | r = -0.894  p = 0.1 |
| **TGF-β3**  **(mRNA)** | r = 0.096  p =0.583 | r = -0.038  p = 0.829 | r = -0.432  p = 0.288 | r = -0.894  p = 0.1 |
| **TGF-β1**  **(H score)** | r = 0.339  p = 0.046 | r = 0.093  p = 0.597 | r = 0.598  p = 0.17 | r = 0.447  p = 0.5 |
| **TGF-β2**  **(H score)** | r = 0.044  p = 0.801 | r = 0.03  p = 0.864 | r = 0.593  p = 0.179 | r = 0.671  p = 0.3 |
| **TGF-β3**  **(H score)** | r = 0.109  p = 0.532 | r = 0.153  p = 0.382 | r = 0.445  p = 0.314 | r = 0.671  p = 0.3 |
| **pSmad2**  **(H score)** | r = 0.042  p = 0.811 | r = 0.184  p = 0.289 | r = 0.371  p = 0.417 | r = 0.447  p = 0.5 |
| **pSmad1/5/8**  **(H score)** | r = -0.012  p = 0.945 | r = 0.138  p = 0.444 | r = 0.898  p = 0.033 | r = 1  p = 0.25 |
| **PDGF-B**  **(mRNA)** | r = 0.119  p = 0.495 | r = 0.031  p = 0.86 | r = -0.259  p = 0.59 | r = 0.224  p = 0.8 |
| **PAI-1**  **(mRNA)** | r = 0.137  p = 0.432 | r = 0.15  p = 0.88 | r = -0.408  p = 0.371 | r = -0.112  p > 0.999 |
| **PAI-1**  **(H score)** | r = 0.007  p = 0.969 | r = 0.055  p = 0.754 | r = 0.176  p = 0.728 | r = -0.056  p > 0.999 |
| **Ki-67 (protein expression)** | r = 0.129  p = 0.474 | r = -0.02  p = 0.913 | r = 0.176  p = 0.728 | r = -0.738  p = 0.333 |
| **TβRIII (H score)**  **endothelial cells** | r = 0.412  p = 0.004 |  | r = 0.818  p < 0.05 |  |

Spearman's rank correlation coefficients (r) and p values (two-tailed t-test) are shown; statistically significant values are highlighted in yellow.

**Supplementary note 1**: Cell culture and reagents

The long-term glioblastoma cell lines (LTC; LN-18, LN-428, LN-319, A172, U87MG, T98G, LN-308 and LN-229) [[1](#_ENREF_1)], and TLA-HEK293T cells (GE Healthcare, Little Chalfont, UK) were cultured in DMEM supplemented with 10% fetal bovine serum (FBS) (VWR Lonza, Leighton Buzzard, UK) and 1% glutamine (Thermo Fisher Scientific, Waltham, MA). The GIC cultures T-325, T-269, ZH-161, S-24 and ZH-305 with maximal passage number 30 [[2](#_ENREF_2)] and the human brain endothelial cell line HCMEC [[3](#_ENREF_3)] were cultured as previously described [[4](#_ENREF_4)]. Human endothelial cell lines (ZHE-459, ZHE-464, ZHE-483-2) isolated from freshly dissected glioblastoma samples were isolated and cultured as previously described [[5](#_ENREF_5)].

Recombinant human (rh) TGF-β2, TGF-β1 and BMP-4 were obtained from R&D Systems (Minneapolis, MN, USA) and SD-208 was obtained from Scios Inc. (Fremont, CA). LTC and GIC were transfected as previously described [[4](#_ENREF_4)]. Stable transfection of cell lines was performed with the expression vectors pcDNA control and pcDNA soluble TβRIII [[6](#_ENREF_6)] and selection of transfected cells was performed with G418 (Millipore Corporation, Billerica, MA). To generate lentivirus-containing supernatant for lentivirally-mediated stable overexpression, HEK293T cells were cotransfected with the vectors LentiORF control (empty backbone, Plasmid 25890 Addgene, Cambridge, MA), LentiORF TβRIII (CCSB-Broad Lentiviral ORF TβRIII, GE Healthcare) and packaging plasmids pMD2.G and pR8.91 using calcium phosphate precipitation. For transient gene silencing siRNA pools containing 3 selected siRNA duplexes (Hs_TGFBR3_1, Hs_TGFBR_2, Hs_TGFBR3_3) and the AllStars Negative Control siRNA (Qiagen, Venlo, Netherlands) were used at 100 nM final concentration. For immunoblot analysis primary antibodies were: anti-Smad2 (3122S), anti-pSmad2 (3108S), anti-Smad5 (9517S), anti-pSmad1/5 (9516S) (all Cell Signaling, Leiden, Netherlands), anti-TβRIII (AF-242-PB, R&D Systems), anti-Actin (sc-1616, Santa Cruz Biotechnology, Inc., Dallas, TX). Visualization of protein bands was accomplished using horseradish peroxidase (HRP)-coupled secondary antibodies (Santa Cruz Biotechnology) and enhanced chemiluminescence (Pierce/Thermo Fisher, Madison, WI). For FACS analysis anti-human TβRIII-PE antibody (FAB242P, R&D Systems) and PE goat IgG isotype control (403004, BioLegend) were used.

**Supplementary note 2:** RT-PCR

Relative quantification of gene expression was determined by comparison of threshold values. [ADP-ribosylation factor 1](http://www.uniprot.org/uniprot/P84077) *(*Arf1) was used as a housekeeping gene. All results were normalized to Arf1 and calculated with the delta(Δ)C_T_ method for relative quantification. Primer sequences were for Arf1 forward 5'-TCC CAC ACA GTG AAG CTG ATG-3', reverse 5'-GAC CAC GAT CCT CTA CAA GC-3' and for TβRIII forward 5’-TAC AGA GAG AGG TCA CAC T-3’, reverse 5’-GTC TTC AGA TGC CAC ACC AG-3’.'

**Supplementary note 3:** ELISA

Cell supernatants were collected from LTC at confluency, from GIC seeded in suspensions of 10^6^/ml and from endothelial cell lines isolated from freshly dissected glioblastoma samples seeded at 2.6 × 10^4^ cells/cm^2^ after 2 days. For LTC and GIC results were expressed corresponding to the cell number at time of supernatant collection. For endothelial cell lines, supernatants were concentrated using Amicon® Ultra-4 centrifugal filter (Millipore Corporation) and results were expressed corresponding to the supernatant protein concentration measured by Pierce® BCA Protein Assay Reagent (Pierce/Thermo Fisher). An ELISA kit for TβRIII from R&D Systems was used for quantification.

**Supplementary note 4**: Animal studies

The standard operating procedures for the animal studies were approved by the Swiss Cantonal Veterinary office under the animal license permission number 80/2013. The care and treatment of all animals were in accordance with the Swiss Federal Law on the Protection of Animals, the Swiss Federal Ordinance on the Protection of Animals and the guidelines of the Swiss confederation. Athymic CD1 nude mice were purchased from Charles River Laboratories (Sulzfeld, Germany).

For intracranial tumor implantation 2 μl of a single cell suspension of 75,000 LN-229 cells in PBS was injected over 2 min into the right striatum using a Hamilton syringe (PS AS needle, 26s gauge, Hamilton, Reno, NV, USA) and the needle was left intracranially for another 3 min.

After initial scoring twice a week, the mice were scored daily and euthanized when reaching a score of 2 either due to development of neurological symptoms or loss of more than 15% of initial weight. For immunohistochemical analysis 3 mice per group were euthanized when any mouse became symptomatic. All brains were collected upon euthanization, embedded in cryomoulds in Shandon Cytochrome yellow (Thermo Fisher Scientific) and frozen on dry ice. Tumor incidences and sizes were determined using hematoxylin and eosin staining of 8 µm thick cryosections cut using a Microm HM560 (Microchom HM560, Thermo Fisher Scientific). Tumor volume was calculated as length multiplied by the width on the largest tumor section multiplied by the tumor depth throughout the brain multiplied by π/6. *Ex vivo* cultures were generated by mechanical dissociation of the tumor in PBS and subsequent culturing in DMEM.

**Supplementary note 5:** Immunohistochemistry

Immunohistochemical staining for TβRIII was performed on a panel of 52 newly diagnosed and 9 recurrent glioblastoma specimens as previously described; data on 49 of these patients have been published previously [[7](#_ENREF_7)]. The normal brain tissue microarray (TMA) comprised tissue samples of 13 individuals. For each sample three 0.6 mm cores were punched from a paraffin block. Tissue samples were taken from 12 hippocampal areas and one temporo-occipital gyrus.

The paraffin sections were incubated over night with TβRIII antibody (AF-242-PB, R&D Systems). Donkey anti-goat HRP antibody (sc2033, Santa Cruz Biotechnologies) was used as secondary antibody and incubated for 30 min at room temperature. The antigen antibody conjugates were detected by staining with diaminobenzidine (Dako, Glostrup, Denmark). The nuclei were stained using hematoxylin and dehydrated before mounting onto coverslips using Eukitt mounting medium (Sigma Aldrich). For immunohistochemistry of mouse brain, cryosections were fixed in 4% formaline for 10 min, pretreated with 3% H_2_O_2_ and blocked in 10% rabbit serum (011-000-002, Jackson Immuno Research, Cambridgeshire, UK) or blocking solution (Candor Biosciences, Wangen, Germany). After blocking, the primary antibodies were applied overnight at 4°C. Primary antibodies were anti-TβRIII (AF-5034-PB, R&D Systems) and anti-pSmad2 (3108, Cell signaling). For pSmad2 donkey anti-rabbit IgG (711-065-152, Jackson Laboratory, Bar Harbor, ME) and for TβRIII detection Histofine simple stain Max PO (414351F, Cosmo Bio, Carlsbad, CA) was used. For the glioblastoma tissue four representative images from the region of highest Ki67 staining were taken. For analysis of TMA staining the whole punch was quantified. The immunostaining was scored using the histo-score (H-score) [[8](#_ENREF_8)], ranging from 0–300 and calculated as the percentage of weakly stained cells plus the percentage of moderately stained cells multiplied by two plus the percentage of strongly stained cells multiplied by three.

**Supplementary note 6:** Immunofluorescence

Immunofluorescence staining of TβRIII and vWF (von Willebrand factor) was carried out on representative fresh frozen glioblastoma sections. Sections were first stained for vWF (A0082, Dako), anti-rabbit FITC (Sigma Aldrich) was used as secondary antibody. In a second step sections were stained for TβRIII (R&D Systems). Donkey anti-goat Alexa594 (Invitrogen) was used as secondary antibody. All sections were mounted in Dako Fluorescent Mounting Medium (Dako). DNA was stained with 4’,6-diamidino-2-phenylindole (DAPI). Pictures were taken with a 40x objective (Leica DMI 6000 inverted microscope, Leica, Wetzlar, Germany).

**Supplementary note 7:** Single-cell real time polymerase chain reaction (scRT-PCR) of reverse transcribed RNA
scRT-PCR was performed with the same glioblastoma tissues and conditions as previously described [[9](#_ENREF_9)]. The glioblastoma tissues were surgically resected and immediately digested with a papain-based dissociation system (Worthington, Lakewood, NJ). Then, leukocytes were depleted by using anti-human CD45-conjugated microbeads (Miltenyi Biotec, Bergisch Gladbach, Germany) and MACS LD columns (Miltenyi Biotec). The single-cell real time polymerase chain reaction–PCR (scRT–PCR) was performed at the Federal Institute of Technology (ETH) Zurich, Department of Biosystems Science and Engineering (D-BSSE) and Genomics Facility in Basel by using C1 Single-Cell Autoprep and BioMark HD instruments (Fludigim, San Francisco, CA, USA). Cells were captured on a C1 Single-Cell Preamp IFC (10–17 μm) using the Fluidigm C1 and capture efficiency was evaluated under an inverted microscope to identify empty sites, sites with debris and multiple cells to exclude them from the final analysis. Preamplified cDNA was generated using the Single Cells-to-CT Kit (Life Technologies, Carlsbad, CA, USA), pooled qPCR primers and FluidigmSTA reagents. Preamplified cDNA was used for high-throughput qPCR measurement of each amplicon using the BioMark HD system with IFC Controller HX (Fluidigm) and 2× SsoFast EvaGreen Supermix with Low ROX (Bio-Rad, Hercules, CA, USA). Single-cell

expression data were collected using the Fluidigm Data Collection software. Cq values were converted to expression levels using the equation Log2Ex = Cq LOD (Limit of Detection) - Cq with a LOD Cq of 25 and data was mean-centered. Mean-centered data was analyzed as previously described [[10](#_ENREF_10), [11](#_ENREF_11)]. Pearson correlation coefficient was calculated for all pairs of expressed genes followed by Benjamini Hochberg correction for multiple testing. Significant correlations were concluded at p< 0.05.

**Supplementary note 8:** Statistical analysis

Data are derived from at least two independent experiments with similar results and results of representative experiments are shown. Means, standard error of the mean (SEM), correlation (r=Spearman’s coefficient or p=two-sided t-test), survival curves (Kaplan-Meier method with log-rank test) and statistical significance (using two-sided unpaired Student’s *t*-test or one sample t-test) were calculated using the software of GraphPad Prism Version 5 (San Diego, CA) and IBM Statistics, Version 25 (SPSS). A p-value of p=0.05 was considered to be statistically significant.

References

1. Weller M (1998) Assessing response to chemotherapy in malignant glioma: the role of steroids. Neurology 50:1196; author reply 1197-8.

2. Le Rhun E, von Achenbach C, Lohmann B, Silginer M, Schneider H, Meetze K, Szabo E and Weller M (2019) Profound, durable and MGMT-independent sensitivity of glioblastoma cells to cyclin-dependent kinase inhibition. Int J Cancer 145:242-253. doi: 10.1002/ijc.32069

3. Weksler BB, Subileau EA, Perriere N, Charneau P, Holloway K, Leveque M, Tricoire-Leignel H, Nicotra A, Bourdoulous S, Turowski P, Male DK, Roux F, Greenwood J, Romero IA and Couraud PO (2005) Blood-brain barrier-specific properties of a human adult brain endothelial cell line. FASEB J 19:1872-4. doi: 04-3458fje [pii] 10.1096/fj.04-3458fje

4. Seystahl K, Tritschler I, Szabo E, Tabatabai G and Weller M (2015) Differential regulation of TGF-beta-induced, ALK-5-mediated VEGF release by SMAD2/3 versus SMAD1/5/8 signaling in glioblastoma. Neuro Oncol 17:254-65. doi: 10.1093/neuonc/nou218

5. Krishnan S, Szabo E, Burghardt I, Frei K, Tabatabai G and Weller M (2015) Modulation of cerebral endothelial cell function by TGF-beta in glioblastoma: VEGF-dependent angiogenesis versus endothelial mesenchymal transition. Oncotarget.

6. Dong M, How T, Kirkbride KC, Gordon KJ, Lee JD, Hempel N, Kelly P, Moeller BJ, Marks JR and Blobe GC (2007) The type III TGF-beta receptor suppresses breast cancer progression. J Clin Invest 117:206-17. doi: 10.1172/JCI29293

7. Frei K, Gramatzki D, Tritschler I, Schroeder JJ, Espinoza L, Rushing EJ and Weller M (2015) Transforming growth factor-beta pathway activity in glioblastoma. Oncotarget 6:5963-77.

8. Goulding H, Pinder S, Cannon P, Pearson D, Nicholson R, Snead D, Bell J, Elston CW, Robertson JF, Blamey RW and et al. (1995) A new immunohistochemical antibody for the assessment of estrogen receptor status on routine formalin-fixed tissue samples. Hum Pathol 26:291-4.

9. Ventura E, Weller M, Macnair W, Eschbach K, Beisel C, Cordazzo C, Claassen M, Zardi L and Burghardt I (2018) TGF-beta induces oncofetal fibronectin that, in turn, modulates TGF-beta superfamily signaling in endothelial cells. J Cell Sci 131. doi: 10.1242/jcs.209619

10. Stahlberg A, Rusnakova V, Forootan A, Anderova M and Kubista M (2013) RT-qPCR work-flow for single-cell data analysis. Methods 59:80-8. doi: 10.1016/j.ymeth.2012.09.007

11. Papa E, Weller M, Weiss T, Ventura E, Burghardt I and Szabo E (2017) Negative control of the HGF/c-MET pathway by TGF-beta: a new look at the regulation of stemness in glioblastoma. Cell Death Dis 8:3210. doi: 10.1038/s41419-017-0051-2

339
