# Supplementary material for: A tumor-promoting role for soluble TβRIII in glioblastoma
Source: Mol Cell Biochem. 2021 Mar 26;476(8):2963–73. doi: 10.1007/s11010-021-04128-y (PMC8263459; doi:10.1007/s11010-021-04128-y)
Supplement: Supplementary file 2 — (PPTX 10466 kb) [file 11010_2021_4128_MOESM2_ESM.pptx]

## Slide 1
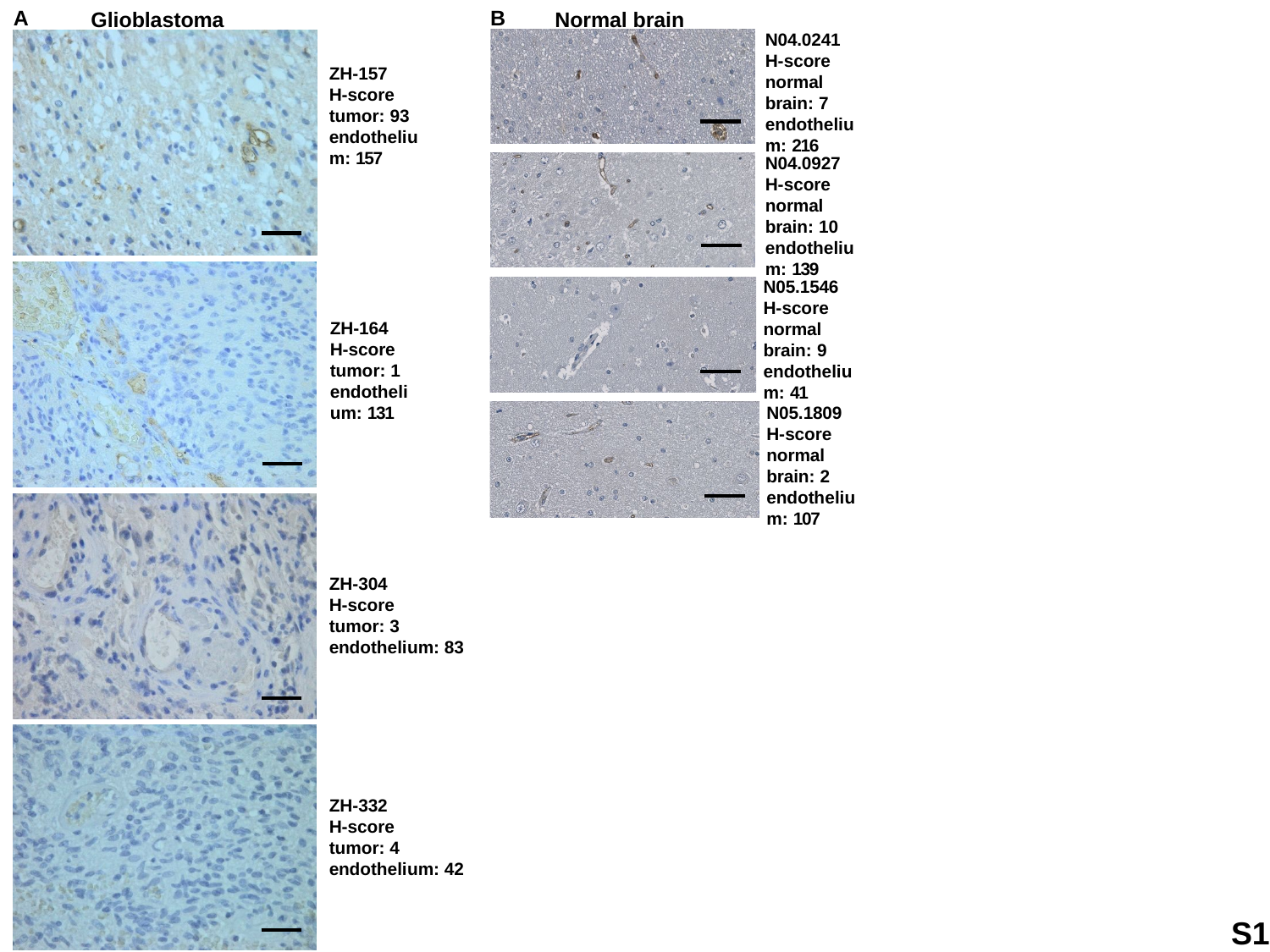

A
B
Glioblastoma
Normal brain
N04.0241
H-score normal brain: 7
endothelium: 216
ZH-157
H-score tumor: 93
endothelium: 157
N04.0927
H-score normal brain: 10
endothelium: 139
N05.1546
H-score normal brain: 9
endothelium: 41
ZH-164
H-score
tumor: 1
endothelium: 131
N05.1809
H-score normal brain: 2
endothelium: 107
ZH-304
H-score
tumor: 3
endothelium: 83
ZH-332
H-score
tumor: 4
endothelium: 42
S1

## Slide 2
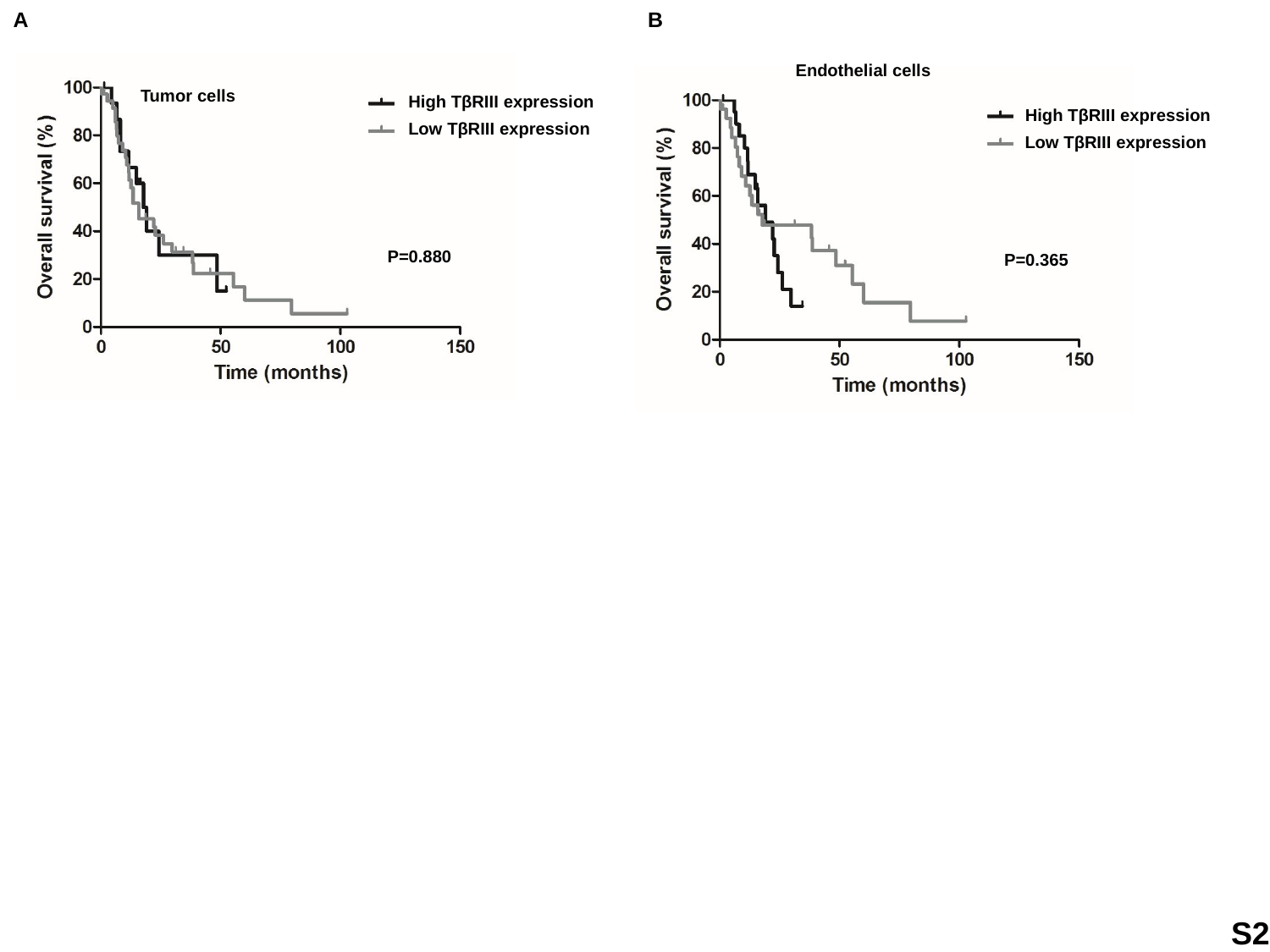

A
B
Endothelial cells
Tumor cells
High TβRIII expression
Low TβRIII expression
P=0.880
High TβRIII expression
Low TβRIII expression
P=0.365
S2

## Slide 3
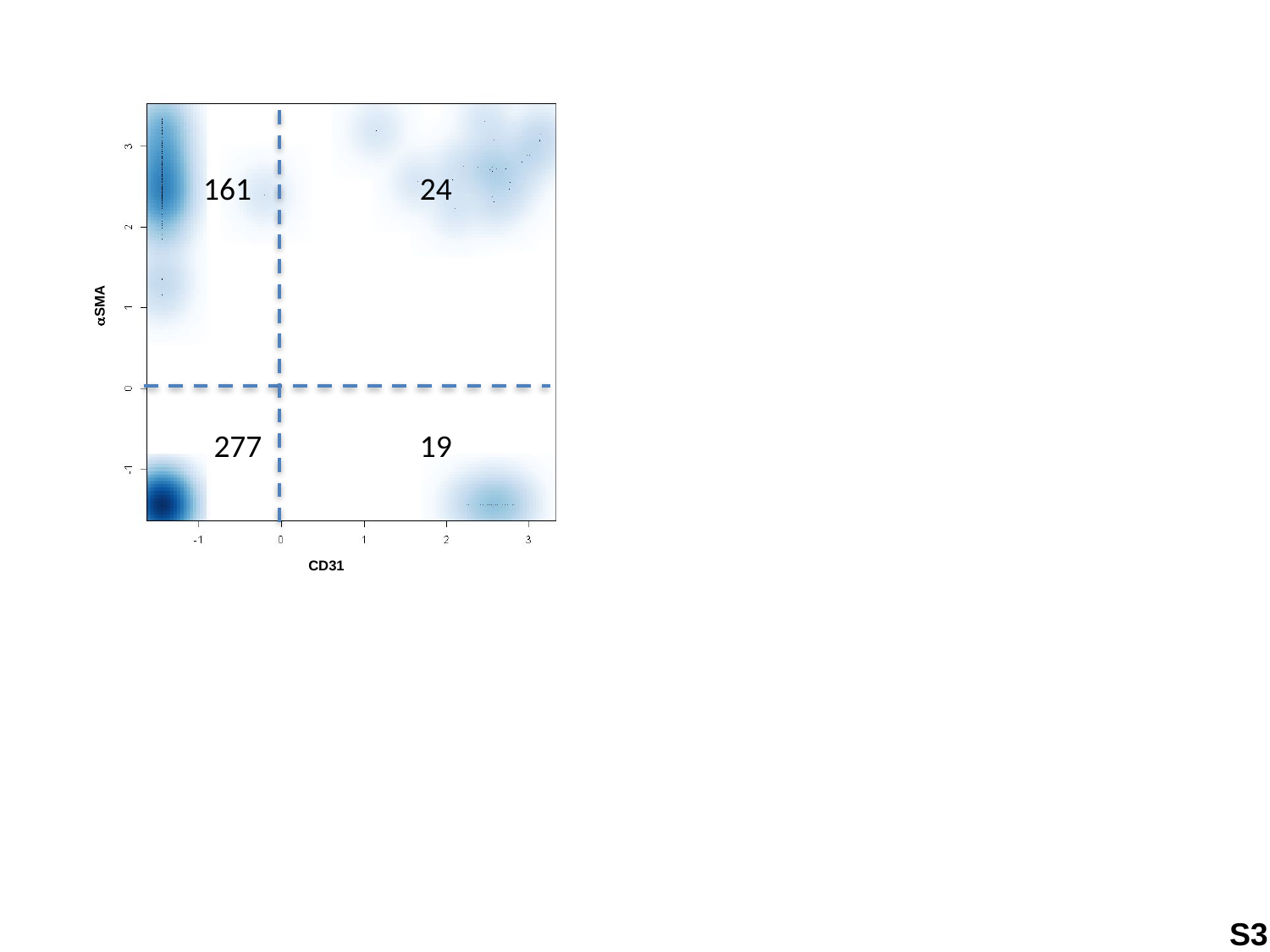

24
161
aSMA
277
19
CD31
S3
